# Integrative Pathway Analysis of Genes and Metabolites Reveals Metabolism Abnormal Subpathway Regions and Modules in Esophageal Squamous Cell Carcinoma

**DOI:** 10.3390/molecules22101599

**Published:** 2017-09-22

**Authors:** Chunquan Li, Qiuyu Wang, Jiquan Ma, Shengshu Shi, Xin Chen, Haixiu Yang, Junwei Han

**Affiliations:** 1 Department of Medical Informatics, Daqing Campus, Harbin Medical University, Daqing 163319, China; 2 College of Bioinformatics Science and Technology, Harbin Medical University, Harbin 150081, China; chenxin00001@163.com (X.C.); yanghaixiu@ems.hrbmu.edu.cn (H.Y.); 3 Department of Computer Science and Technology, Heilongjiang University, Harbin 150080, China; jiquanma@126.com (J.M.); 2001043@hlju.edu.cn (S.S.)

**Keywords:** metabolic pathway, network, ESCC

## Abstract

Aberrant metabolism is one of the main driving forces in the initiation and development of ESCC. Both genes and metabolites play important roles in metabolic pathways. Integrative pathway analysis of both genes and metabolites will thus help to interpret the underlying biological phenomena. Here, we performed integrative pathway analysis of gene and metabolite profiles by analyzing six gene expression profiles and seven metabolite profiles of ESCC. Multiple known and novel subpathways associated with ESCC, such as ‘beta-Alanine metabolism’, were identified via the cooperative use of differential genes, differential metabolites, and their positional importance information in pathways. Furthermore, a global ESCC-Related Metabolic (ERM) network was constructed and 31 modules were identified on the basis of clustering analysis in the ERM network. We found that the three modules located just to the center regions of the ERM network—especially the core region of Module_1—primarily consisted of aldehyde dehydrogenase (ALDH) superfamily members, which contributes to the development of ESCC. For Module_4, pyruvate and the genes and metabolites in its adjacent region were clustered together, and formed a core region within the module. Several prognostic genes, including GPT, ALDH1B1, ABAT, WBSCR22 and MDH1, appeared in the three center modules of the network, suggesting that they can become potentially prognostic markers in ESCC.

## 1. Introduction

Esophageal cancer is one of the most common cancer types worldwide, ranking eighth in order of occurrence and sixth as the leading cause of cancer mortality [1]. The 5-year overall survival for advanced esophageal cancer is less than 10% [2]. In recent decades, esophageal squamous-cell carcinoma (ESCC), which is the main type of esophageal cancer, remains a threat in China [3,4,5]. Aberrant metabolism is one of the main driving forces in the initiation and development of most of cancer, including prostate cancer, ESCC, etc. [6]. For example, the phenomenon, known as the Warburg effect, refers to the preference of cancer cells to metabolize glucose by aerobic glycolysis [7]. Evidence that cancer is mainly a metabolic disease enables us to identify biomarkers for diagnosis and prognosis, as well as the pathological mechanism of many cancers from the perspective of metabolism [8]. However, the detailed etiology of ESCC—apart from the Warburg effect—is still largely unknown.

With the development of high-throughput testing technology, systems biology research strategies are attracting much attention. The rapid technological advancements in obtaining high-throughput omics data, combined with the development of pathway analysis methodologies, have recently enhanced our ability to study metabolism on a genome-wide scale [8,9]. Many ‘omics’ technologies, including microarrays, RNA-seq, and gas chromatography mass spectrometry (GC-MS), are available for identifying potentially differential genes, non-coding RNA, and metabolites in cancer [9,10,11]. Metabolic pathway analysis has become a popular approach to understanding these differential genes and metabolites because metabolic pathways contain them, and their dysfunction plays important roles in cancer [9,12].

Here, we performed integrative pathway analysis of gene and metabolite profiles. By obtaining multiple gene expression profiles and metabolite profiles, differential genes and metabolites in ESCC were identified for each data set. To effectively identify ESCC-related metabolic pathways, the differentially expressed genes in at least four datasets were used as locating key regions of pathways. Through the cooperative use of differential genes, differential metabolites and their positional importance information in pathways, metabolic subpathways associated with ESCC were then identified by the Subpathway-GM software tool. Furthermore, a global ERM network was constructed by merging genes and metabolites in each significant subpathways. Thirty-one modules were identified on the basis of clustering analysis in the ERM network. Analysis of ESCC-related metabolic network predicted key genes and metabolites in ESCC, some of which could potentially become prognostic markers in ESCC.

## 2. Results

### 2.1. Identification of Metabolic Subpathways Associated with ESCC

We identified differentially expressed genes in six data sets of gene expression profiles of ESCC. We found that many genes were differentially expressed in multiple gene expression profiles and number of the differential genes in more than four data sets significantly decreased (Figure S1). To ensure accuracy and stability of differential genes, we used differentially expressed genes in at least four data sets as ESCC-related common differential genes, and ultimately identified a total of 3107 genes.

Compared with transcriptomic technology, which can provide thousands of differential molecules for single study, most current metabolomic technologies usually only analyze a small fraction of the entire metabolome, and dozens of metabolites can be identified as differential due to technological limitations. It is preferable to identify these metabolites in the profiles with their corresponding studies, because they often depend on a priori knowledge [8]. Compared with transcriptomic technology, metabolomic technologies tend to identify fewer differential molecules, but with a greater degree of accuracy. Thus, we adopted a lenient strategy for merging differential metabolites from the results of seven experimental studies. Notably, if a metabolite appeared in at least one study, the metabolite was considered to be an ESCC-related differential metabolite for further pathway analysis. Finally, a total of 115 unique differential metabolites associated with ESCC were obtained.

Through integrating 3107 ESCC-related genes with 115 metabolites, we subsequently identified key abnormal regions of metabolic pathways using the Subpathway-GM method [9]. Subpathway-GM can provide an accurate level of subpathway identification by integrating information from genes and metabolites, along with their positions and cascade regions within the given pathway [9]. Because the number of differential genes related to ESCC was obviously greater than that of the differential metabolites, identification of abnormal regions of Subpathway-GM mainly depended on the differential genes. Adopting a strict strategy that ESCC-related differential genes must appear in at least four data sets can ensure a more accurate identification of abnormal regions. Furthermore, the differential metabolites associated with ESCC can help to identify abnormal regions with metabolite abnormalities. Finally, we used the Subpathway-GM method with FDR < 0.01 to identify 39 significant metabolic subpathways from among all metabolic pathways, based on 3107 differential genes and 115 differential metabolites (Table 1).

### 2.2. Local Region Analysis of Subpathways Revealed Important Functional Genes and Metabolites

**Figure 1:**
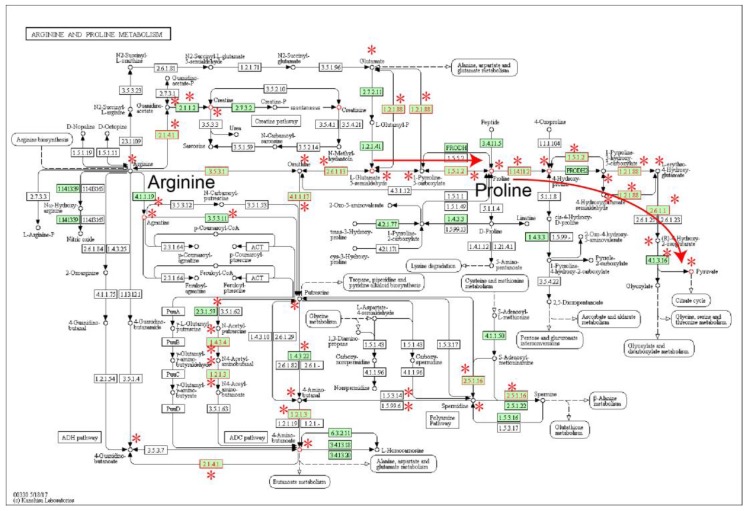
The ‘Arginine and proline metabolism’ pathway, in which the differential genes and metabolites of ESCC are annotated. Nodes near asterisk symbols belong to the subpathway region (path:00330_1). Enzymes (rectangular nodes) mapped by differential genes and metabolites (circle nodes) are shown with red node labels and borders.

The most significant subpathway (path:00330_1) was ‘arginine and proline metabolism’ (Figure 1). Proline, a core metabolite of the pathway, was highly differential in ESCC. Moreover, the key region where 1-Pyrroline-5-carboxylate and l-erythro-4-Hydroxyglutamate are converted by pyrroline-5-carboxylate reductase (PYCR, 1.5.1.2) to proline (red arrow region in Figure 1) was closely related to ROS production in ESCC [13]. Compared with arginine metabolism (left region in Figure 1), genes and metabolites in the proline metabolism region were more abnormal in ESCC (right in Figure 1).

The second-most significant subpathway (path:00280_1) was ‘valine, leucine and isoleucine degradation metabolism’ (Figure S2). Subpathway-GM yielded an FDR value of 1.32 × 10^−5^. Most of genes in the pathway were differentially expressed in ESCC, and were located in all three sub-regions of the pathway. Valine, leucine and isoleucine were located at the starting region of three sub-regions of the pathway, and all of them were differential in ESCC. Surprisingly, the pathway and genes within it were not reported to be associated with ESCC. Only one metabolite, aline, was reported to be able to help result in high diagnostic capacity for ESCC metastasis [14]. However, we found that 20 genes in the ‘valine, leucine and isoleucine degradation metabolism’ subpathway were differentially expressed in at least four data expression profiles, and six metabolites were abnormal in ESCC. On the basis of the differential expression of most genes and core metabolites in the pathway, our integrative analysis might find a novel ESCC-related pathway.

**Figure 2:**
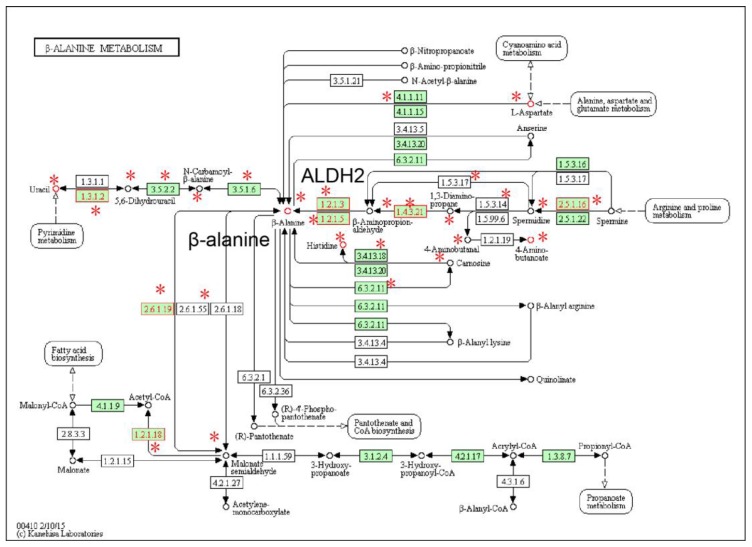
The ‘beta-Alanine metabolism’ pathway, in which the differential genes and metabolites of ESCC are annotated. Nodes near asterisk symbols belong to the subpathway region (path:00410_2). Enzymes (rectangular nodes) mapped by differential genes and metabolites (circle nodes) are shown with red node labels and borders.

The third-most significant subpathway (path:00410_2) was ‘beta-Alanine metabolism’ (Figure 2). Subpathway-GM yielded an FDR value of 3.38 × 10^−5^. Five metabolites, including β-alanine, aspartate, uracil, 4-aminobutanoate and histidine, were differential in ESCC. Although they were not reported in ESCC, β-alanine, a center metabolite of the pathway, displayed potential anti-cancer effects in renal and cervical tumor cells [15]. Histidine was previously reported by us to be associated with prostate cancer [9]. Gene ALDH2 (EC:1.2.1.3) is highly associated with the development of ESCC [16]. Other ALDH superfamily members are also highly associated with ESCC risk in Asian populations [16,17,18]. Alcohol intake is an important risk factor that contributes to the development of ESCC in Asian and other populations [19]. A high degree of differential genes and metabolites within β-alanine metabolism, such as β-alanine and ALDH2 suggested, that the ‘beta-Alanine metabolism’ pathway might be a new pathway that is highly associated with ESCC, as predicted by our integrative analysis.

**Figure 3:**
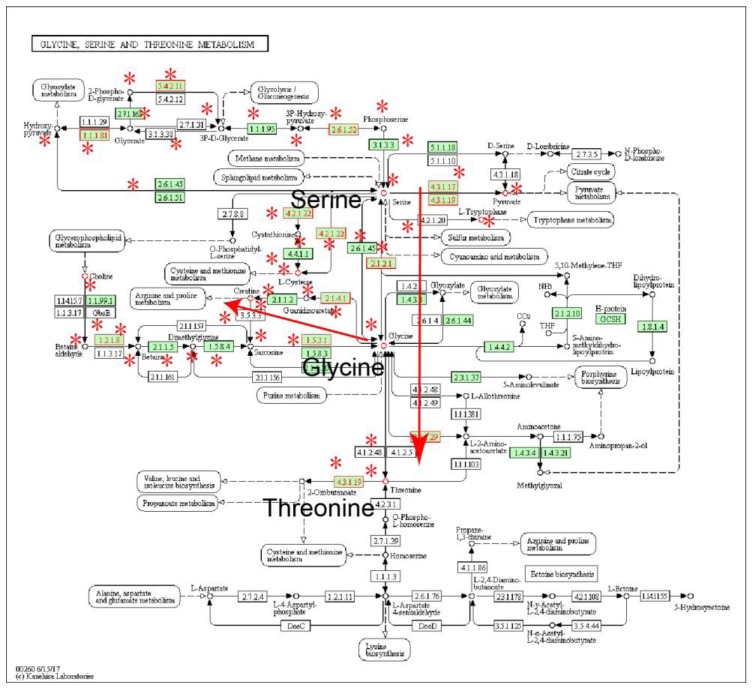
The ‘glycine, serine and threonine metabolism’ pathway, in which the differential genes and metabolites of ESCC are annotated. Nodes near asterisk symbols belong to the subpathway region (path:00260_1). Enzymes (rectangular nodes) mapped by differential genes and metabolites (circle nodes) are shown with red node labels and borders.

The fourth-most significant subpathway (path:00260_1) belonged to ‘glycine, serine and threonine metabolism’ (Figure 3). Subpathway-GM yielded an FDR value of 4.60 × 10^−5^. All three core metabolites of the pathway, including glycine, serine and threonine, were differential in ESCC. In the core path of ‘glycine and serine metabolism’ (red arrow region in Figure 3), serine hydroxymethyltransferase (SHMT; EC:2.1.2.1) can convert glycine to serine, and the reaction is reversible. SHMT affects gene methylation and DNA synthesis, and is closely related to the development and progression of cancer. SHMT1 1420C/T genotype can significantly reduce susceptibility to ESCC [20]. Subpathway-GM used distance similarity information between genes and metabolites to identify the key subpathway region. The center metabolic region of the ‘glycine, serine and threonine metabolism’ pathway was closely related to glycine [13]. Glycine can be converted by pyrroline-5-carboxylate reductase (PYCR, EC:1.5.1.2) to creatine (red arrow region in Figure 3). Both the protein-coding gene PYCR and the metabolite creatine were differential in ESCC, and the metabolite crosstalks with the most significant ‘arginine and proline metabolism’ pathway, indicating that glycine, creatine, arginine and proline metabolism plays an important role in ESCC.

In addition, multiple other subpathways have been proved to be cancer-related pathways. For example, the fifth-most significant subpathway (path:00010_1) was the ‘glycolysis/gluconeogenesis’ pathway, which is highly associated with many different kinds of cancer. During the 20th century, the Warburg effect was presented, which describes the phenomenon where, in cancer cell metabolism, cancer cells consume glucose and acidify their environment with lactate [9]. Another few subpathways, such as ‘pyruvate metabolism’, ‘citrate cycle (TCA cycle) metabolism’, and ‘arachidonic acid and purine metabolism’, were also often reported to play a role in many cancers. Taken together, our integrative analysis has effectively identified multiple known and novel subpathways associated with ESCC.

### 2.3. Network Analysis Revealed Important Functional Modules and Genes in ESCC

**Figure 4:**
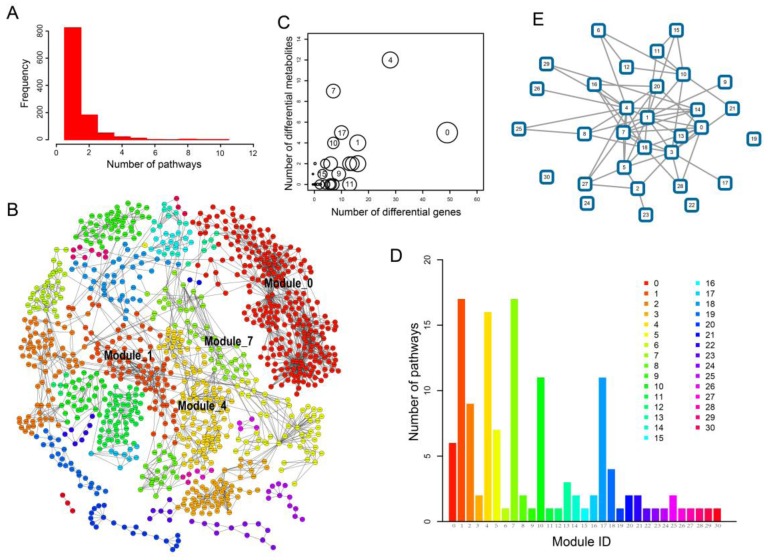
Analysis of ESCC-Related Metabolic network. (**A**) Distribution of genes/metabolites with respect to number of pathways they appears at; (**B**) The result visualization of the clustering analysis of the network using the ModuLand method. The same color nodes belong to the same module. Use of module color is the same as Figure 4D; (**C**) The bubble plot of modules. X-axis represents number of differential genes in modules. Y-axis represents number of differential metabolites in modules. Size of circle represents number of genes and metabolites in modules; (**D**) The number of pathways associated with modules, which refers to how many pathways are associated with the genes and metabolites in the corresponding module; (**E**) The network for crosstalk between modules.

**Figure 5:**
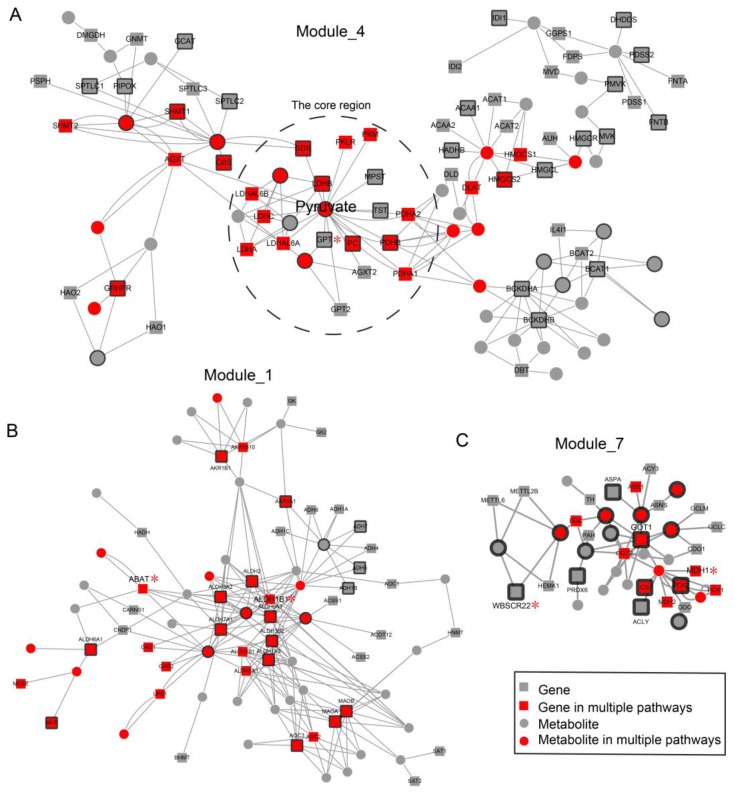
The representative modules of ESCC-related metabolic network. (**A**) Module_4. (**B**) Module_1. (**C**) Module_7. Nodes with black borders are the differential genes or metabolites. Nodes near asterisk symbols represents ESCC prognostic genes.

We found that many genes and metabolites perform functions in multiple subpathways/pathways. Notably, 204 (19.28%) genes and 66 (18.59%) metabolites appeared in at least two of the ESCC-related pathways (Figure 4A). Pyruvate was annotated in up to ten pathways. ALDH7A1, a member of subfamily 7 in the aldehyde dehydrogenase gene family, appeared in up to ten pathways, and was abnormally expressed in ESCC. Other members in the aldehyde dehydrogenase gene family, including ALDH2, ALDH9A1, ALDH3A2, ALDH1A3 and ALDH3B2, also appeared in multiple pathways and were differentially expressed in ESCC. This suggested that the significant metabolic pathways in ESCC might be closely associated with each other. To better explore functional genes and metabolites between pathways in ESCC from a global perspective, we constructed an ESCC-Related Metabolic (ERM) network by merging all genes and metabolites in the significant subpathways. The resulting network was composed of 1037 nodes (696 genes and 341 metabolites) and 2468 edges (Figure 4B). Clustering analysis using the ModuLand method showed that nodes in the ERM network were clustered into 31 modules (Figure 4B) with crosstalk relationships (Figure 4E). Gene ontology analysis of modules was performed for each module (Table S1). Module_4 and Module_0 contained the highest number of differential metabolites and differential genes in ESCC, respectively (Figure 4C). Interestingly, up to 17 (54.83%) modules were composed of genes/metabolites from distinct pathways, suggesting that many genes and metabolites from different pathways crosstalk as a union cluster. For example, the genes and metabolites in Module_1 (Figure 5B) came from up to 17 pathways (Figure 4D). Moreover, 40.74% genes/metabolites in the module function in multiple pathways. Module_4 and Module_7 (Figure 5A,C) were also composed of genes/metabolites coming from more than 15 pathways (Figure 4D). These three modules were located just right of the center regions of the ERM network (Figure 4B), suggesting the importance of Module_1, Module_4 and Module_7 in ESCC. Furthermore, gene ontology analysis of these modules also showed that they performed key metabolic functions, such as the pyruvate metabolic process, gluconeogenesis and response to drug (Table S1).

Module_4 was located in the center region of the ERM network, and contained the highest number of differential metabolites in ESCC (Figure 4B,C). This indicates that the molecules in Module_4 play a core role in ESCC. In the module, pyruvate, one of the most important metabolites, is located in the central position, and 18 genes interact with it, 7 of which are differentially expressed in ESCC, including LDHB, SDS, PDHB, MPST, TST, PC and GPT (Figure 5A). Pyruvate and the genes and metabolites in its adjacent region were clustered together and form a core region within the module (Figure 5A). Lactate dehydrogenase B (LDHB) catalyzes the conversion of lactate to pyruvate. LDHB activity is necessary for cancer cell proliferation not only in oxidative cancer cells, but also in glycolytic cancer cells [21]. The targeting LDHB activity inhibits the proliferation of cancer cells preferentially to normal differentiated cells [21].

Another module located in the center region of the network is Module_1. The gene and metabolites in the module appeared in up to 17 metabolic pathways. The core region of Module_1 mainly consisted of aldehyde dehydrogenase (ALDH) superfamily members, of which multiple genes, such as ALDH7A1, ALDH2, ALDH9A1, ALDH3A2, ALDH1A3 and ALDH3B2, were differentially expressed in ESCC (Figure 5B). The ALDH superfamily members are major enzymes involved in the alcohol-metabolizing pathways, and are highly associated with ESCC risk [16,17,18,22,23,24]. Alcohol intake is an important risk factor that contributes to the development of ESCC in Asian and other populations [19]. ALDH7A1 was located in up to ten pathways and was abnormally expressed in ESCC. ALDH7A1, one of the ALDH superfamily members, degrades and detoxifies acetaldehyde generated by alcohol metabolism, and has been associated with development and prognosis of multiple cancers [22]. A joint analysis showed that drinkers with both the ADH1B and ALDH2 risk alleles had a fourfold increased risk of ESCC compared to drinkers without these risk alleles [16,17,18,22,23,24].

### 2.4. ESCC-Related Prognostic Genes Mainly Located in Peripheral Regions of Network

Prognostic genes are important molecules for cancer treatment. Therefore, we mapped the prognostic genes, which were obtained from the gene expression profiles of 119 ESCC patients with clinical follow-up, to the ESCC-related metabolic network. The result showed that 47 prognostic genes appeared in the ERM network (Figure S3). Most prognostic genes (72.34%) were non-differentially expressed genes, and were not located in the center of the network. For example, Module_0 contained the greatest number of prognostic genes (12 genes). However, Module_0 is not located in the center of the network. In contrast, the center modules, i.e., Module_1, Module_4 and Module_7, each contained fewer than three prognostic markers (2, 1, and 2 prognostic genes, respectively), showing that prognostic genes are mainly located in peripheral regions of the network. A recent study showed that prognostic genes in many cancers tend not to be topologically important genes present to large extents in the network [25], suggesting that prognostic genes tend to be located in peripheral regions of network. To further test this in the ERM network, the degree of prognostic genes was measured. The results showed that the degree of prognostic genes was significantly lower than other genes (*p* value = 0.0053; Wilcoxon rank-sum test). Because the degree of nodes only measures the local structure of nodes in a network (that is, nearest neighbors), we further calculated the betweenness centrality of nodes able to measure a more global network feature. Similarly, the betweenness centrality of prognostic genes was also lower than that of other genes (*p* value < 10 × 10^−8^), suggesting that prognostic genes are located in topologically peripheral regions of network. Prognostic genes might be different from many important functional cancer genes, which are often located in topologically important areas. This can help us to understand network features of prognostic biomarkers in ESCC and identify new prognostic biomarkers according to these network features.

### 2.5. Prognostic Genes that Appeared in the Center Modules of the Network

**Figure 6:**
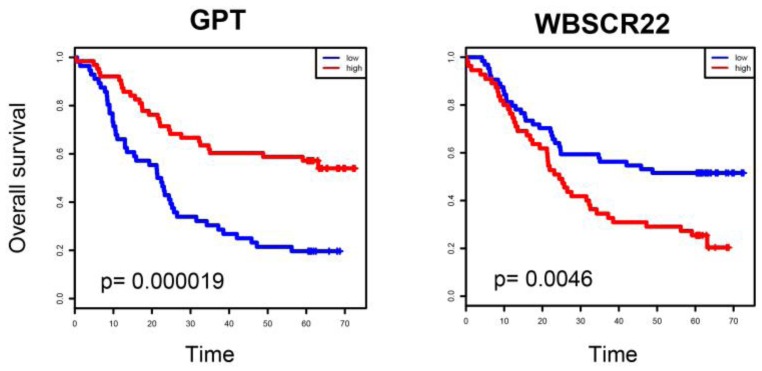
Kaplan-Meier curves of ESCC patients with either higher or lower expression of GPT and WBSCR22.

Although ESCC prognostic genes were located in topologically peripheral regions of the ESCC-related metabolic network, five prognostic genes, including AMD1, DNMT1, GPX6, GSTM5 and MDH1, appeared between modules. The mutation of all these genes was highly associated with cancer initiation and progression [26,27,28,29,30], suggesting importance of these genes in ESCC. When we focused on genes in modules, several prognostic genes, including GPT, ALDH1B1, ABAT, WBSCR22 and MDH1, appeared in the center modules of the network, i.e., Module_4, Module_1 and Module_7 (Figure 5). Moreover, GPT and WBSCR22 were highly statistically significant in survival analysis (*p* = 0.000019 and 0.0046, respectively) (Figure 6). Gene GPT was the most significant prognostic gene in the ERM network. Patients with high GPT expression had a significantly shorter survival time than those with low expression (*p* = 0.000019) (Figure 6). GPT was located in the alanine, aspartate and glutamate metabolism pathway, and belonged to Module_4, a center region of the network associated with pyruvate. GPT encodes cytosolic alanine aminotransaminase, also known as glutamate-pyruvate transaminase 1, which catalyzes the reversible transamination between alanine and 2-oxoglutarate to generate pyruvate and glutamate. Glutamate-pyruvate transaminase 1 was not reported to be associated with cancer, although it is routinely used as a biomarker of liver injury caused by alcohol. Alcohol intake is an important risk factor that contributes to the development of ESCC in Asian and other populations [16,17,18,19,22,23,24]. This indicated that GPT might play an important role in ESCC, and become a new prognostic gene of cancer.

## 3. Discussion

Integrative metabolic pathway analysis of genes and metabolites can better help to interpret the underlying biological phenomena. Through analyzing six gene expression profiles and seven metabolite profiles of ESCC, the differential genes and metabolites in ESCC were identified and then used to locate 39 significant subpathway regions of metabolic pathways, in consideration of the joint use of differential genes, differential metabolites and the positional importance of genes and metabolites. To ensure the accuracy and stability of ESCC-related differential genes, genes that were differentially expressed in at least four datasets were used to locate key regions of pathways. This strict strategy can ensure a more accurate identification of ESCC-related differential genes. The limitations of metabolite identification technology mean that fewer differentially expressed metabolites are detected compared with differentially expressed genes, which may result in pathway analysis strategies tending to ignore metabolite information. However, metabolites may be located in important positions of pathways. Subpathway-GM takes into account the importance of metabolites in locating and evaluating subpathways. We found that, using our integrative pathway analysis pipeline, multiple subpathways, such as those belonging to ‘arginine and proline metabolism’ and ‘glycine, serine and threonine metabolism’, were associated with ESCC. Some novel subpathways, such as ‘valine, leucine and isoleucine degradation metabolism’ and ‘beta-Alanine metabolism’, were identified.

Furthermore, we found that ESCC-related metabolic pathways might be closely associated with each other by sharing ESCC-related metabolites and genes such as pyruvate and ALDH2. Genes that code proteins and enzymes further perform metabolic functions, and might play an important role in diseases [8,31]. We thus performed a global ESCC-related metabolic network analysis by merging genes and metabolites in each significant subpathway. Analysis of ESCC-related metabolic networks identified 31 modules and predicted key genes and metabolites in ESCC. Modules represent the important regions of the network [32,33]. We found that Module_1, Module_4 and Module_7 were located in the center region of the ERM network (Figure 4B). Pyruvate, one of the most important metabolites, is located in the central position of Module_4, and 7 genes that interact with it were differentially expressed in ESCC, including LDHB, SDS, PDHB, MPST, TST, PC and GPT. The core region of Module_1 mainly consisted of aldehyde dehydrogenase (ALDH) superfamily members, of which multiple genes, such as ALDH7A1 and ALDH2, were differentially expressed in ESCC. The ALDH superfamily members are major enzymes involved in alcohol-metabolizing pathways, and are highly associated with ESCC risk [16,17,18]. Prognostic analysis showed that 47 prognostic genes appeared in the network. Several prognostic genes, including GPT, ALDH1B1, ABAT, WBSCR22 and MDH1, appeared in the center modules of the network, i.e., Module_1, Module_4 and Module_7. GPT was located in the alanine, aspartate and glutamate metabolism pathway, and belonged to Module_4, a center region of the network associated with pyruvate. Our findings establish the utility of integrative bioinformatic analyses to identify functional metabolism abnormal subpathway regions and modules in ESCC.

## 4. Materials and Methods

### 4.1. Metabolites Highly Related with ESCC

Differential metabolites were directly obtained from the results of several metabolomic experimental studies, including Xu et al. [1], Liu et al. [3], Wu et al. [34], Ma et al. [35], Hasim et al. [36], Jin et al. [14] and Wang et al. [37]. Table S2 provides detailed information about ESCC-related metabolic profile data. The metabolites were extracted from these papers and converted to KEGG compound IDs. Finally, a total of 115 unique differential metabolites associated with ESCC were obtained.

### 4.2. Gene Expression Profiles and Differential Genes Related with ESCC

Six sets of gene expression profiles of ESCC were obtained from the Gene Expression Omnibus (GEO) database (http://www.ncbi.nlm.nih.gov/geo/), including GSE17351, GSE20347, GSE23400, GSE29001, GSE32424 and GSE29968. The first four sets of gene expression profiles were detected using microarray technology, and the other two were detected using RNA-sequencing technology. Table S3 provides detailed information about ESCC-related gene expression profile data. For microarray datasets, a gene was considered to be differentially expressed when it was seen to be significant using the SAM method at a significance level of 0.05 (FDR < 0.05) [38]. For RNA-sequencing datasets, a gene was considered to be differentially expressed when FDR < 0.05 using the DESeq (version 1.14.0) method [4].

### 4.3. Integrative Pathway Analysis Pipeline

For each gene expression dataset, the differential genes were identified using DESeq for RNA-seq data and SAM for microarray data, because the two methods have been used by many studies, and have proved to be very effective. Other methods could be used instead. For example, the LIMMA method can handle both RNA-seq and microarray data [39]. To ensure accuracy and stability of the subpathway identification, we used differentially expressed genes in at least four datasets as ESCC-related common differential genes. Compared with transcriptomic technology, metabolomic technologies tend to identify less, but are more representative of differential molecules. Thus, we considered a metabolite as an ESCC-related differential metabolite if it appeared in at least one study (that is, it was significantly differential in at least one study). We used Subpathway-GM to identify metabolic subpathways [9]. Compared with other popular pathway analysis methods [12], Subpathway-GM was able to locate key subpathway regions accurately via positional information of differential genes and metabolites within pathways [9]. Briefly, Subpathway-GM firstly annotates differential genes and metabolites to metabolic pathways. Secondly, according to the shortest path between the mapped genes/metabolites, subpathways were located and mined for each pathway. If the shortest path length among differential nodes (e.g., differential genes or metabolites) was shorter than n + 1 (the default value n = 5 was used in the paper), then these nodes and other non-signature nodes in the shortest path were merged as a subpathway. Finally, the statistical significance of subpathways was evaluated by using hypergeometric test. Through using representative differential metabolites and depending on differentially expressed genes with high accuracy and stability, Subpathway-GM tends to identify the key regions representative of entire pathways.

We input ESCC-related common differential genes and metabolites to Subpathway-GM in order to locate significant subpathway regions in ESCC. Then, the ERM network was constructed based on the results for these significant subpathway regions. Notably, all genes and metabolites in the significant subpathways with FDR < 0.01 were extracted and considered as nodes in the ERM network. Thus, nodes in the network were genes or metabolites that appeared in at least one significant metabolic subpathway in ESCC. The edges in the ERM network were added according to reaction relationship between genes and metabolite in those significant subpathways with FDR < 0.01. An edge between two nodes in the network will be linked if there exists one edge between them within at least one of all significant subpathways. We used Cytoscape (http://www.cytoscape.org/) [40] to visualize the network and analysis the properties of the network.

### 4.4. Identification of Network Modules

The network was imported into the cytoscape [40]. A plug-in, ModuLand, was used to identify modules [33]. The resulting modules were displayed using ModuLand, which can color each node of the network. Moreover, the hierarchical layers of a network can be constructed through linking edges between modules. The tool can determine key nodes between two or multiple modules.

### 4.5. Survival Analysis

The ESCC gene expression profiling from 119 Chinese patients with follow-up information (minimum of 5 years) was obtained from GEO database (GSE53624) [41]. The clinical characteristics of the patients can be obtained from GEO (https://www.ncbi.nlm.nih.gov/geo/query/acc.cgi?acc=GSE53624) or the original paper (http://gut.bmj.com/content/gutjnl/suppl/2014/02/12/gutjnl-2013-305806.DC1/gutjnl-2013-305806supp_table1.pdf). For the dataset, the lncRNA and protein-coding gene expression level from 119 paired tumor-normal samples were measured by Agilent human lncRNA + mRNA array. We only used protein-coding gene expression data. The microarray data was log 2-scale transformed. To reduce the influence of heterogeneity between different patients, the expression value of tumor minus normal was used, which was consistent with the original paper for the dataset [41]. For each protein-coding gene, patients were divided into either a high-risk group or a low-risk group on the basis of mean value of this gene expression level in all samples [42]. The relationship between gene expression levels and prognosis of ESCC patients was performed by Kaplan-Meier analysis and statistical significance was assessed using the log-rank test on R 2.15.2 framework [9,42]. Gene with *p* value < 0.05 were considered as prognostic genes.

## Figures and Tables

**Table 1:** The 39 significant subpathways identified by Subpathway-GM.

Subpathway Id	Pathway Name	*p* Value	FDR
path:00330_1	Arginine and proline metabolism	4.93 × 10^−12^	4.19 × 10^−10^
path:00280_1	Valine, leucine and isoleucine degradation	3.12 × 10^−7^	1.32 × 10^−5^
path:00410_2	beta-Alanine metabolism	1.19 × 10^−6^	3.38 × 10^−5^
path:00260_1	Glycine, serine and threonine metabolism	2.16 × 10^−6^	4.60 × 10^−5^
path:00010_1	Glycolysis/Gluconeogenesis	1.45 × 10^−5^	0.00021
path:00270_1	Cysteine and methionine metabolism	1.59 × 10^−5^	0.00021
path:00250_2	Alanine, aspartate and glutamate metabolism	1.99 × 10^−5^	0.00021
path:00250_1	Alanine, aspartate and glutamate metabolism	2.05 × 10^−5^	0.00021
path:00604_1	Glycosphingolipid biosynthesis—ganglio series	2.80 × 10^−5^	0.00026
path:00531_2	Glycosaminoglycan degradation	3.50 × 10^−5^	0.00029
path:00240_1	Pyrimidine metabolism	5.39 × 10^−5^	0.00041
path:00340_1	Histidine metabolism	7.77 × 10^−5^	0.00053
path:00052_2	Galactose metabolism	8.18 × 10^−5^	0.00053
path:00520_1	Amino sugar and nucleotide sugar metabolism	0.00011	0.00067
path:00562_1	Inositol phosphate metabolism	0.00011	0.00067
path:00620_1	Pyruvate metabolism	0.00015	0.00081
path:00640_1	Propanoate metabolism	0.00028	0.0014
path:00230_1	Purine metabolism	0.00031	0.0014
path:00630_1	Glyoxylate and dicarboxylate metabolism	0.00040	0.0017
path:00360_1	Phenylalanine metabolism	0.00041	0.0017
path:00600_1	Sphingolipid metabolism	0.00046	0.0018
path:00532_1	Glycosaminoglycan biosynthesis—chondroitin sulfate	0.00048	0.0018
path:00510_1	N-Glycan biosynthesis	0.00063	0.0023
path:00480_1	Glutathione metabolism	0.00073	0.0025
path:00030_1	Pentose phosphate pathway	0.0010	0.0035
path:00100_4	Steroid biosynthesis	0.0011	0.0038
path:00020_1	Citrate cycle (TCA cycle)	0.0013	0.0042
path:00062_2	Fatty acid elongation	0.0014	0.0043
path:00350_2	Tyrosine metabolism	0.0014	0.0043
path:00561_1	Glycerolipid metabolism	0.0016	0.0046
path:00460_1	Cyanoamino acid metabolism	0.0020	0.0056
path:00053_3	Ascorbate and aldarate metabolism	0.0028	0.0074
path:00770_2	Pantothenate and CoA biosynthesis	0.0031	0.0077
path:00900_2	Terpenoid backbone biosynthesis	0.0031	0.0077
path:00603_1	Glycosphingolipid biosynthesis—globo series	0.0032	0.0077
path:00650_2	Butanoate metabolism	0.0032	0.0077
path:00601_1	Glycosphingolipid biosynthesis—lacto and neolacto series	0.0034	0.0078
path:00760_1	Nicotinate and nicotinamide metabolism	0.0043	0.0094
path:00590_1	Arachidonic acid metabolism	0.0043	0.0094

## Supplementary Materials

Supplementary materials are available online. Figure S1: Distribution of genes with respect to number of data sets. X-axis represents the number of data sets. Y-axis represents number of genes that appear at the corresponding number of datasets. For example, >4000 out of all genes appears in only one of datasets. Figure S2: The ‘valine, leucine and isoleucine degradation metabolism’ pathway where the differential genes and metabolites of ESCC were annotated. Nodes near asterisk symbol belong to the subpathway region (path:00280_1). Enzymes (rectangular nodes) mapped by differential genes and metabolites (circle nodes) are shown with red node labels and borders. Figure S3: Kaplan-Meier curves of ESCC patients with either higher or lower expression of 47 prognostic genes. Table S1: Gene ontology annotation of modules. Table S2: Detail information about ESCC-related metabolic profile data. Table S3: Detail information about ESCC-related gene expression profile data.
